# Does Net-Speak Experience Interfere With the Processing of Standard Words? Evidence From Net-Speak Word Recognition and Semantic Decisions

**DOI:** 10.3389/fpsyg.2020.01932

**Published:** 2020-08-07

**Authors:** Jingjun Chen, Sudan Huang, Rong Luo

**Affiliations:** ^1^Department of Psychology, Hunan University of Science and Technology, Xiangtan, China; ^2^Chenzhou No.1 People’s Hospital, Chenzhou, China

**Keywords:** net-speak, Chinese word, net-speak word, recognition, semantic decision task

## Abstract

The relationship between Chinese net-speak use and traditional literacy has rarely been discussed in the literature. In this study, we conducted two experiments to explore the effects of net-speak experience on word recognition and semantic decisions. A sample of senior middle school students was divided into a high-experience group and a low-experience group according to the students’ net-speak experience, and the Go/No-Go task (Experiment 1) was adopted to investigate the differences between the two groups in the recognition of pure net-speak words and standard words. The results showed that the response time (RT) of participants was longer for pure net-speak word recognition than for standard word recognition. In addition, for both types of words, the recognition RT of participants in the high-experience group was shorter than that of participants in the low-experience group, but the accuracy (ACC) of pure net-speak word recognition was higher. Taking dual semantic net-speak words with both net-speak meaning and traditional meaning as priming stimuli and standard words related to the two meanings as targets, a semantic decision task (Experiment 2) was used to explore the differences between the two groups in judgments of the semantic relationship between the target words and priming words. The results showed that the decision ACC of participants in the high-experience group for both kinds of meaning-related words was significantly higher than that of participants in the low-experience group. The decision RT of participants in the high-experience group was slightly shorter for net-speak meaning-related words than traditional meaning-related words. The decision RT and ACC of participants in the low-experience group for both kinds of meaning-related words were equivalent. This shows that for Chinese teenagers, net-speak use may not disturb the processing of standard words; on the contrary, it may enhance processing.

## Introduction

“Net-speak” is an alternative to “Netlish,” “Weblish,” “Internet language,” “cyberspeak,” “electronic language,” “electronic discourse,” “interactive written discourse,” “computer-mediated communication” (CMC), and other more cumbersome locutions (Crystal, 2006). The representative words in net-speak are formed through the widespread use of SMS and IM among teenagers and have been referred to as “textisms.” They are generally abbreviated or slang forms and quite different from standard words in terms of spelling and grammar rules. Other terms are used to refer to these orthographically and unconventional word forms, such as “SMS language,” “text language,” “SMS speak,” “textspeak,” and “textese” (Lieke, 2013). Waldron et al. (2015) concluded that there are more than 10 different types of “textisms,” and most of these types use different forms of orthographic or phonological abbreviations, such as contractions (e.g., msg for message), phonological abbreviations (e.g., thru for through), initialisms (e.g., omg for oh my God), shortenings (e.g., goin for going), single letters (e.g., u for you), combined letters (e.g., 2day for today), accent stylizations (e.g., gonna for going to) and misspellings (e.g., rember for remember). Additional forms, such as repeating letters (soooo for so) and typographic symbols (<3 for love), are non-abbreviations.

Since textisms are very different from conventional English words or phrases in terms of morphology, orthography and spelling, there are concerns that long-term use of textisms may adversely affect children’s standard literacy. Many studies have explored the relationship between children’s use of textisms and their standard literacy, but most of the findings have shown that children who text more and use more textese obtain higher scores on assessments of literacy skills (Waldron et al., 2015; Blom et al., 2017). These literacy skills include word reading (Plester et al., 2009; Coe and Oakhill, 2011), phonological awareness (Plester et al., 2009; Wood et al., 2011), and spelling (Plester et al., 2008; Bushnell et al., 2011; Wood et al., 2011). Researchers believe this may be because the forms of textisms are mostly based on phonological information rather than symbolic information, revealing an understanding of ‘conventional’ letter-sound correspondences and orthographic rules in English, even though the resultant spellings are viewed as unconventional (Plester et al., 2009). Only when children have a good command of conventional English can they participate in this game, which in turn gives children more opportunities to create written language and communication, thus promoting their traditional literacy skills. Most textisms seem to be variants of allo-graphic homophonous synonyms of conventional words. This means that for experienced net-speakers, there is a close connection between English net-speak words and standard words in the mental lexicon; thus, the cognitive processing of the two may have some similarities as well as some differences. This supposition was initially confirmed in a series of studies on English textism language processing. Ganushchak et al. (2010) used a lexical decision task to compare the processing of shortcuts (net-speak words, such as ezy) and pseudo-shortcuts (non-net-speak words, such as eze), in which participants were asked to make word/non-word judgments while their ERPs (event-related potentials) were recorded. The results showed that shortcuts were categorized as non-words more slowly than pseudo-shortcuts and that the N400 amplitude (270–500 ms) was more negative for pseudo-shortcuts than for shortcuts. Ganushchak et al.’s (2010) results suggested that shortcuts activated stored lexical representations of their basic words and that their semantic processing characteristics were therefore similar to those of standard words. Ganushchak et al. (2012) used a masked priming lexical decision paradigm to compare the priming effects of shortcuts and their corresponding words (conventional forms). The shortcuts or corresponding words with the same meaning were the priming words, and the semantic-related words were the target stimuli. In the case of single-word priming materials (gr8/great—good), the two types of primes produced a similar priming effect with 57 or 1000 ms stimulus-onset asynchrony (SOA). In the case of phrase priming materials, with 57 ms SOA, corresponding word primes (see you—goodbye) facilitated the target processing more than shortcut primes (cu—goodbye). With 1000 ms SOA, this priming advantage of corresponding word primes disappeared, and there was priming from component-related word primes (*see* you—*look*) but not from shortcut primes (cu—look). The authors concluded that the meanings of shortcuts can be retrieved from the mental lexicon without being translated into their corresponding words. These results indicated that in the mental lexicon of experienced net-speakers, at least for a conventional single word and its online form, the link between orthography and semantics may be of equal distance.

However, researchers (Head et al., 2013a) employed a masked priming lexical decision task to examine the priming effects of conventional words (TEXT), subset words (TXT, an abbreviated form of a complete word with a letter omitted) and non-words (GRFT) and found that subset primes produced significantly faster and more accurate responses to target probes than non-words. Additionally, conventional word primes produced faster and more accurate responses (BLESS—bless) to target probes than subset words (blss—bless) and non-words. The questionnaire revealed that those who reported more experience with net-speak benefited more from net-speak primes than those who reported less experience. This study showed that experience with net-speak helped to strengthen the link between the orthography of net-speak words and their meaning, but the link was not as strong as that between the orthography of conventional words and their meaning. In another study, Head et al. (2012b) used the Go/No-Go task to explore the reaction time (RT) and accuracy (ACC) of college students in detecting “text” in a series of conventional words and “txt” in subset words. A 9-min detection task was divided into four continuous 2.25 min periods. The researchers calculated the mean RT of the participants in each period and found that the mean RT increased linearly from periods 1–4 for the subset detection task but not for the word detection task. This means that subset (text-speak) processing may indeed be more cognitively demanding and susceptible to fatigue than word processing.

In the literature on sentence reading, Farrell and Lyddy (2012) found that when participants had to monitor for a vibration around their waist while reading a story, longer RTs and fewer correct vibration detections occurred when the story was written with textisms than when the story used correctly spelled words. The results implied that reading the net-speak text imposed a greater cognitive load than reading the correctly spelled text. Eye movement studies involving reading text showed that even for experienced net-speakers, the reading time for textisms was longer than that for conventional words, with more fixations (Sinéad et al., 2015). Perea et al. (2009) also found that it took longer to read sentences with textisms than conventional sentences. The results showed that there was a reading cost for individuals who were highly expert in SMS language. Neuro-scientific studies revealed that the processing of net-speak words in sentences required more cognitive resources and produced an obvious N400 delay effect. Berger and Coch (2010) employed ERPs to compare the N400 effects of processing semantically inconsistent words in conventional English sentences with those in net-speak sentences. They found that a significant N400 component could be induced in both conventional English sentences and net-speak sentences. However, the peak latency was delayed to 500–700 ms for the net semantically incongruent sentences, and the N400 component had a longer duration than in conventional semantically incongruent sentences. The result is similar to the processing difference between native language and second language, which means that processing semantic information for sentences including textisms involves more neurocognitive resources. One study (Head et al., 2013b) used fNIRS to explore the processing of net-speak and found that compared with the processing of standard English, net-speak processing involved significant activation in the right prefrontal cortex. The authors suggested that this increased activity in the right hemisphere may signify a compensatory effort in processing text-speak sentences, which would indicate that the cognitive cost of net-speak processing is higher.

In conclusion, the above studies on the cognitive processing of English net-speak words show that the processing of textisms is similar to that of standard words for experienced net-speakers; for example, the two kinds of words equally activate semantic processing between 270 and 500 ms (Ganushchak et al., 2010) and have equivalent semantic priming effects (Ganushchak et al., 2012). However, additional studies have found that even for participants experienced with net-speak, the processing of net-speak words requires more cognitive effort than the processing of conventional words (Perea et al., 2009; Berger and Coch, 2010; Farrell and Lyddy, 2012; Head et al., 2012a, 2013a; Sinéad et al., 2015). Researchers (Head et al., 2012a) have suggested that this may be related to certain orthographic differences between net-speak words and conventional words, and individuals may need to additionally activate standard orthography when processing net-speak words, because “no user is likely to be more proficient with text-speak relative to correctly conventional speak, if they do so, it could induce more errors, especially with time-on-task” (pp. 110).

**FIGURE 1:**
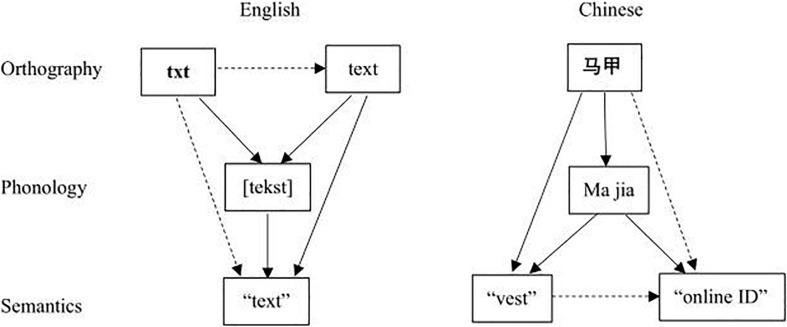
Lexical access model for English and Chinese net-speak words.

For net-speak words in English, their heteromorphy with conventional words (such as g8-great) determines the processing differences at the orthographic level. However, there is no such relationship between Chinese net-speak words and conventional words. Chinese net-speak words have a variety of forms, such as symbol graphics (:-) means a smiling face), phonetic translations of foreign words (e.g., “copy” is translated into “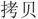 Kao bei”), novel words (e.g., “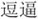 Dou bi” means ridiculous people, which is a new coinage), and letter abbreviations (BF means boyfriend), and these can be considered pure net-speak words with only net-speak meaning. A form of Chinese net-speak very different from textism also exists, namely, the neologism (“old word with new meaning”), which is created when a net-speak meaning is given to a standard word. Most Chinese net-speak words are neologisms, which consist of two subclasses: homophone substitutions and homographs. Homophone substitutions involve a standard word replacing another word based on their homophonic relationship (e.g., “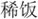 Xi fan” replaces “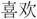 Xi huan,” that is, “rice congee” possesses the meaning of “love” online). Therefore, the net-speak meaning is completely different from the traditional meaning. The net-speak meaning of homographs is generated through the metaphorizing of the traditional meaning (e.g., the original meaning of “
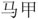
” is vest, but the net-speak meaning is identity). In homophone substitution, the net-speak meaning of a word is unrelated to its conventional meaning, but the net-speak and conventional words are homonymous. With homographs, the two meanings are related to each other to some extent, so the word is polysemous. Obviously, these two kinds of words are ambiguous, so we can call them dual semantic net-speak words. Therefore, the processing of dual semantic net-speak words is different from that of English textisms, and its particularity may be reflected at the semantic level (see Figure 1). In Figure 1, the solid arrows represent the confirmed activation paths, and the dotted arrows represent the speculated activation paths based on previous studies. For a conventional English word, its phonology and semantics are directly activated by orthography, and its orthography also indirectly activates semantics through the mediator of phonology. For textism, its informal orthography activates phonology first and then semantics, and it is unclear whether standard orthography and semantics are directly activated by informal orthography. Similarly, in Chinese, there are inherent links among the conventional orthography, phonology and semantics of a dual semantic net-speak word. However, in the online context, whether the net-speak meaning is activated directly by orthography or by the mediator of conventional semantics needs to be determined.

A recent study on the processing of ambiguous Chinese words found that polysemy and homonymy were represented similarly in the mental lexicon, and their multiple meanings were represented as separate entries (Shen and Li, 2016). This means that the two meanings of a net-speak word with dual semantics can be accessed separately. Semantic access is influenced by language experience, context and meaning frequency. Therefore, for those with more experience with net-speak, the net-speak meaning may be accessed more quickly than the traditional meaning due to the higher frequency of use of the net-speak meaning. In addition, due to the similarities in orthography, for dual semantic net-speak words, the experience of using one meaning may not only promote the cognitive processing of another meaning but also produce competition between the two meanings in an unknown context. This provides an opportunity to investigate whether net-speak experience is conducive to the processing of conventional words. Although a recent study (Zhang et al., 2019) suggested that the use of net-speak enhanced creative problem solving, few studies have paid close attention to the relationship between net-speak experience and the processing of conventional Chinese words.

Qingbai et al. (2017) used the classical semantic incongruity paradigm and ERP technology to explore the semantic processing of Chinese net-speak words by taking sentences as the unit of study. They found that for both net-speak language and standard language, a more negative N400 component was induced in the incongruity condition, but this N400 component was characterized by a delayed peak latency and an extended duration in net-speak compared with standard language. Qingbai et al. (2017) suggested that these results reflected the participants’ relatively low fluency in the use of net-speak and implied the continuation of semantic cognitive conflict. In the semantic congruity condition, net-speak words elicited a more negative novel N400 and a more positive late component (LPC) than standard words. The novel N400 reflected the recognition of the novel semantic information of net-speak words, while the LPC reflected semantic integration and novel semantic association. Qingbai et al. (2017) explained that the result was due to the fact that the participants had some familiarity with net-speak words, but net-speak words were not processed automatically. Although this study suggests that more cognitive resources are needed for the semantic processing of net-speak words, it is difficult to directly explain whether this cognitive cost is caused by the lack of experience with net-speak or the competition between the traditional meaning and net-speak meaning of dual semantic net-speak words.

This study represents the first attempt to discuss the influence of net-speak experience on the processing of standard Chinese words. First, we use the Go/No-Go task (Experiment 1) to test the RT and ACC of participants with different net-speak experience in recognizing different pure net-speak words and standard words. The main purpose is to investigate whether high-experience users have better recognition of conventional words than low-experience users. Because experienced net-speak users may have higher language sensitivity and creativity, we hypothesize that they are better at recognizing both net-speak words and conventional words.

Second, we use a semantic decision task (Experiment 2) to investigate the RT and ACC of net-speak users with different experience levels in deciding whether standard words and their meanings are related to one of the meanings of dual semantic net-speak words. The main purpose is to investigate whether high-experience users are better at deciding both meanings than low-experience users to illustrate that net-speak experience does not exert a negative influence on the comprehension of traditional meanings. Because the words share orthography, our basic hypothesis is that, for a net-speak word with both a traditional meaning and net-speak meaning, net-speak experience strengthens the link between its net-speak meaning and orthography but does not weaken the link between its traditional meaning and orthography.

## Experiment 1

### Method

#### Participants

One hundred five students from class Nos. 315 and 316, grade one, of a senior middle school in Zixing city, Hunan Province, China, were recruited as participants. These students were in key classes, which means that they all passed rigorous entrance tests and had achieved excellent academic results. They were invited to complete a questionnaire about their net-speak experience (see section “Material”). Based on their scores, 30 students in the top 30% were assigned to the high-experience group, and the bottom 30% were assigned to the low-experience group. There was a significant difference between the two groups in terms of net-speak experience (*t* = −15.767, *p* < 0.001, Cohen’s *d* = 0.90). The 60 participants were in the 14–16 age range (*M* = 14.92, *SD* = 0.53; male = 24, female = 36). All participants had normal or corrected-to-normal vision.

#### Material

***The Adolescent Familiarity with and Use of Net-speak Questionnaire*** (AFUNQ, Appendix A) was used to investigate familiarity with and usage of net-speak and to split the original sample into two net-speak experience groups, namely, the high-experience group and the low-experience group. The 100 net-speak words, including 44 pure net-speak words and 56 dual semantic net-speak words, used in the questionnaire came from *http://wangci.net/* and are currently the most commonly used and most popular words. For each word, the participants chose the following options: “I have never heard of that,” “I have heard of it but I don’t know the exact meaning,” “I know the meaning but I haven’t used it,” “Occasionally used,” “Infrequently used,” and “Frequently used.” A six-point rating scale was adopted. The Cronbach’s alpha of the questionnaire was 0.980. Taking self-reported frequency of internet use as the criterion, we estimated the criterion validity of the AFUNQ and found that the Pearson’s correlation coefficient between frequency of internet use and the AFUNQ score was very significant (*r* = 0.265, *p* < 0.001).

#### Word Stimuli

Forty net-speak words, 40 conventional words and 80 pseudo-words were chosen as stimuli (Appendix B). Thirty-five net-speak words were selected from the AFUNQ. A total of 308 senior middle school students self-reported familiarity with these net-speak words, and the participants in Experiments 1 and 2 were included as reporters. The 35 pure net-speak words with the highest scores and five additional pure net-speak words from the real-time update of http://wangci.net/ were used as the experimental stimuli and consisted of 25 two-character words, 12 three-character words and 3 four-character words. The conventional words were selected from among the top 3000 words with the highest frequency in the Modern Chinese frequency dictionary (Institute of Language Teaching and Research, 1986), which was compiled based on a corpus of 1.3 million words. These words were distributed in the frequency range of 72–2585 (mean = 1168.50) and had a similar number of strokes and characters as the net-speak words. Additionally, we also created 80 orthographically legal pseudo-words made up of common characters, and they had neither online nor traditional meanings.

#### Design

We employed a 2 × 3 two-factor mixed design with net-speak experience (between subjects: high versus low) and word type (within subjects: net-speak word, standard word and pseudo-word) as independent variables. The dependent variables were decision ACC and RT.

#### Procedure

**FIGURE 2:**
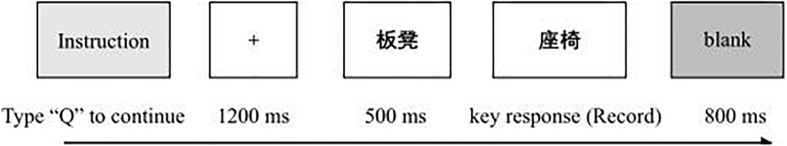
Example of a trial in Experiment 1.

The participants were tested at individual cubical stations. They were seated 50 cm in front of video display terminals (409 mm × 255 mm, 1024 × 768 screen resolution, 75 Hz refresh rate) that were mounted at eye level. Stimuli presentation and RT and ACC recordings were performed by personal computers running E-prime Professional 2.0. All the stimuli were presented in the center of the computer screen. The fixation “+” was presented in red, and the words were presented in black in Courier New 34. A total of 160 words, including net-speak words, standard words and pseudo-words, were randomly presented, and each word appeared once. Participants were instructed to respond to the semantic stimuli by pressing the space bar and to withhold responses to nonsense pseudo-words. Responses occurring within 1800 ms after the onset of the pseudo-words were recorded as errors of commission, and critical stimuli not responded to were recorded as errors of omission. The experimental task lasted approximately 10 min. The core process is shown in Figure 2.

### Results

We first organized the experimental data before analyzing them. In the RT list for correct responses, we deleted cases 3 SD above or below the average; then, we inspected the ACC in the same way and deleted cases 3 SD below the average. Ultimately, we deleted five cases, and 55 valid cases remained.

#### ACC of Participants’ Judgments

The means and standard deviations of participants’ ACC in the different tasks are presented in Table 1.

**TABLE 1:** ACC of participants for three kinds of words (*M, SD*).

	**Net-speak word**	**Standard word**	**Pseudo-word**
Low-experience group (*n* = 27)	0.945 (0.056)	0.987 (0.017)	0.884 (0.118)
High-experience group (*n* = 28)	0.984 (0.022)	0.986 (0.025)	0.875 (0.112)

**FIGURE 3:**
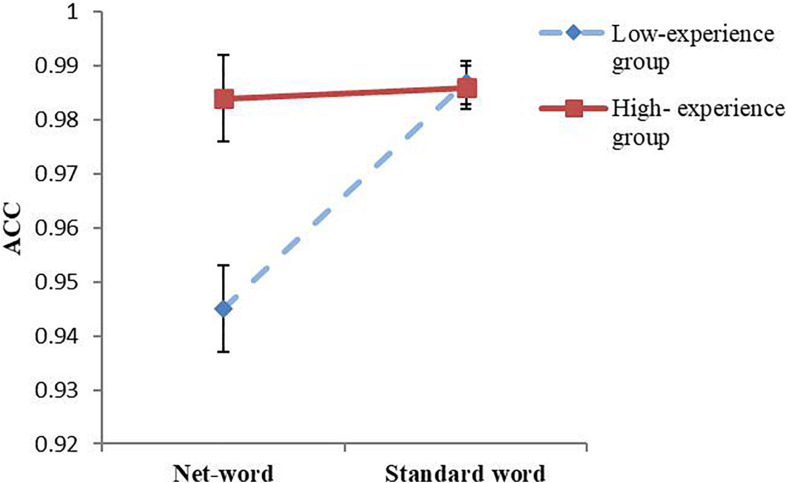
ACC of the two groups for two kinds of words (error bars depict the standard errors of the mean).

Repeated-measures ANOVA was used to analyze the effects of the independent variables on ACC. The results showed that the main effects of word type and net-speak experience were significant (*F*_1,53_ = 14.94, *p* < 0.001, $\eta_{\text{p}}^{2}$ = 0.220; *F*_1,53_ = 7.02, *p* = 0.011, $\eta_{\text{p}}^{2}$ = 0.117), and there was a significant interaction between the two variables (*F*_1,53_ = 12.29, *p* = 0.001, $\eta_{\text{p}}^{2}$ = 0.187) (Figure 3).

The results of simple-effect tests indicated that the ACC of the high-experience group for net-speak words was significantly higher than that of the low-experience group (*F*_1,53_ = 11.51, *p* = 0.001, $\eta_{\text{p}}^{2}$ = 0.178), but for standard words, there was no difference (*F*_1,53_ = 0.08, *p* = 0.774). This means that net-speak experience promotes the processing of net-speak words and does not hinder the processing of standard words.

Additionally, we compared the ACC of pseudo-word decisions between the two groups and found that there was no significant difference (*t* = 0.31, *p* = 0.762). However, the pseudo-word ACC was significantly lower than the ACC for the other two types of words, both in the high-experience group and the low-experience group (*F*_2,52_ = 13.21, *p* < 0.001, $\eta_{\text{p}}^{2}$ = 0.337; *F*_2,52_ = 25.42, *p* < 0.001, $\eta_{\text{p}}^{2}$ = 0.485). We speculate that pseudo-words are more like pure net-speak words and that participants have the tendency to pursue speed, so they pressed the space key instead of inhibiting the response, making errors of commission common.

#### RT of Participants’ Judgments

The participants’ RT means and standard deviations (ms) for the different tasks are presented in Table 2.

**TABLE 2:** RT of participants for two kinds of words (*M, SD*).

	**Net-speak word**	**Standard word**
Low-experience group (*n* = 27)	685.22 (88.35)	653.12 (96.26)
High-experience group (*n* = 28)	644.29 (57.91)	610.88 (63.17)

We explored the effects of the independent variables on RT by repeated-measures ANOVA. The results indicated that there were significant main effects of experience group and word type (*F*_1,53_ = 4.41, *p* = 0.041, $\eta_{\text{p}}^{2}$ = 0.077; *F*_1,53_ = 22.09, *p* < 0.001, $\eta_{\text{p}}^{2}$ = 0.294), but there was no significant interaction between the two variables (*F*_1,53_ = 0.009, *p* = 0.925).

The results showed that the RT in the two groups to net-speak words was longer than the RT to standard words, and the responses of the high-experience group to both kinds of words were significantly faster than those of the low-experience group.

### Discussion

In Experiment 1, we examined the RT and ACC of participants using the Go/No-Go task to explore the effects of net-speak experience on the recognition of pure net-speak words and standard words. The results revealed that the high-experience group responded more quickly to net-speak words and standard words than the low-experience group. In addition, the ACC for net-speak words was higher than that for standard words, but the ACC for standard words showed no significant difference between the two groups. This implies that participants who are more familiar with net-speak words activate more quickly not only the meanings of pure net-speak words but also the meanings of standard words.

Head et al. (2013a) found that the priming effect of net-speak words on relevant standard words was more obvious in participants with more net-speak experience than in those with less experience. Therefore, for the participants highly experienced in net-speak, it seems that there are fixed and powerful links between the orthography of net-speak words and their meanings. For experienced users of net-speak (such as college students), the activation of net-speak words in the mental lexicon is similar to that of standard words (Ganushchak et al., 2010), and the meaning retrieval of net-speak words is direct rather than involving the conversion of standard words (Ganushchak et al., 2012). In the present study, we found that net-speak experience indeed benefitted the recognition of net-speak words.

More importantly, our findings are similar to those in the English context—children who use textism more frequently performed better on literacy-related assessments (Blom et al., 2017). Our results also suggested that net-speak experience may promote the processing of standard words. However, there may be another possibility: Plester et al. (2009) have pointed out that people experienced in net-speak themselves may have a high level of language sensitivity and ability, which is the reason why they are interested in net-speak words and use them creatively. Although the participants were all excellent students selected based on their scores on the entrance examination, we actually did not collect information about their language sensitivity and ability. Therefore, the superiority of the high-experience group in responding to standard words may not be due solely to net-speak experience—it may also depend on linguistic sensitivity and ability. Perhaps there was reciprocal causation between language level and net-speak use. Regardless, we found that the use of net-speak at least did not have a negative impact on the users’ understanding of standard words.

## Experiment 2

Experiment 1 revealed that the high-experience group exhibited better processing of net-speak words and standard words than the low-experience group. This raises another key question: For a net-speak word with both net-speak meaning and traditional meaning, which meaning is activated first? Therefore, in Experiment 2, we used net-speak words with double meanings as stimuli to detect semantic competition in the mental lexicon.

### Method

#### Participants

One hundred one students were selected from class Nos. 317 and 318, grade one, of a senior middle school in Zixing city, Hunan Province, China. Participants in the experiment were those that ranked in the top 30% and bottom 30% of the scores on the AFUNQ (see Experiment 1). All participants had normal or corrected-to-normal vision and ranged in age between 14 and 16 (*M* = 14.95, *SD* = 0.43; male = 28, female = 32). There was a significant difference between the two groups of students in net-speak experience according to a *t*-test (*t* = −14.787, *p* < 0.001, Cohen’s *d* = 0.89).

#### Design

We employed a 2 × 2 two-factor mixed design with net-speak experience (between subjects: high versus low) and word type (within subjects: traditional meaning-related words and net-speak meaning-related words) as independent variables. The dependent variables were ACC of judgment and RT of correct judgment.

#### Material

Thirty dual semantic net-speak words were used as the priming stimuli. An example is “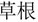 cao gen,” the traditional meaning of which is grass root and the net-speak meaning of which is a person at the bottom of society. The stimuli comprised three single-character words, 24 two-character words, 2 three-character words and 1 four-character word. Twenty-eight of these words were selected from the 56 dual semantic net-speak words in the AFUNQ, and their scores were the top scores of the 56 words assessed by 308 students (see section “Material,” Experiment 1); another two words were from real-time updates of http://wangci.net/. Sixty related words were the paired targets, including 30 net-speak meaning-related words (such as “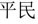 ping min,” which means civilian) and 30 traditional meaning-related words (such as “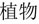 zhi wu,” which means plant) (Appendix C). Fifty-four students from another class (No. 312) rated the 30 net-speak words on a seven-point scale and rated their relevance to the two meanings. The results showed that the degree of association with traditional meaning-related words was between 3.96 and 5.70 (Kendall’s coefficient *W* = 0.062, χ^2^ = 96.931, *df* = 29, *p* < 0.001), and the degree of association with net-speak meaning-related words was between 2.57 and 6.61 (Kendall’s coefficient *W* = 0.152, χ^2^ = 238.431, *df* = 29, *p* < 0.001). A paired-sample *t*-test (*t* = 0.201, *p* = 0.144) showed that there was no significant difference between the ratings of the net-speak meaning-related words (*M* = 5.259, *SD* = 0.973) and those of the traditional meaning-related words (*M* = 4.814, *SD* = 1.285).

#### Procedure

**FIGURE 4:**
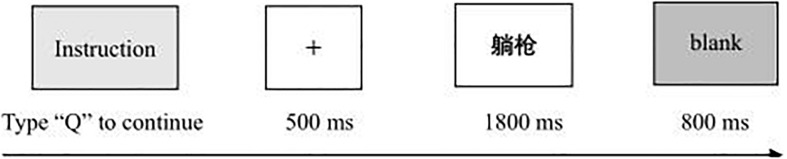
Example of a trial in Experiment 2.

The semantic decision task was adopted in this experiment. The computer randomly presented the priming stimulus words for 500 ms, and the target word would then appear. The task of the participants was to determine whether the target word was related to the traditional meaning or net-speak meaning of the priming stimulus. The target word disappeared after participants pressed the button or 1800 ms after it appeared. Half of the participants pressed the F-key to indicate net-speak meaning and the J-key to indicate traditional meaning, while the other half pressed the opposite keys. Each of the 60 word pairs was randomly presented twice for a total of 120 responses. The E-prime program recorded the RT and ACC. The experiment lasted for approximately 10 min, and the participants were given small gifts after completing the experiment. The core process is shown in Figure 4.

### Results

We first organized the experimental data before analyzing them. We deleted one case because the data were 3 SD below the ACC average. Ultimately, 59 valid cases remained.

#### ACC of Participants’ Judgments

The participants’ means and standard deviations of ACC in the different tasks are presented in Table 3.

**TABLE 3:** ACC of participants for two kinds of words (*M, SD*).

	**Traditional meaning-related word**	**Net-speak meaning- related word**
Low-experience group (*n* = 30)	0.697 (0.168)	0.683 (0.174)
High-experience group (*n* = 29)	0.747 (0.172)	0.768 (0.145)

Repeated-measures ANOVA was used to analyze the effects of the independent variables on ACC. The results showed that the main effect of net-speak experience was almost significant (*F*_1,57_ = 3.16, *p* = 0.081, $\eta_{\text{p}}^{2}$ = 0.053), and there were no other significant effects. The ACC of the high-experience group was higher than that of the low-experience group.

#### RT of Participants’ Judgments

The participants’ RT means and standard deviations in the different tasks are presented in Table 4.

**TABLE 4:** RT of participants for two kinds of words (*M, SD*).

	**Traditional meaning-related word**	**Net meaning-related word**
Low-experience group (*n* = 30)	763.72 (279.49)	765.50 (281.92)
High-experience group (*n* = 29)	765.85 (222.43)	729.69 (234.38)

**FIGURE 5:**
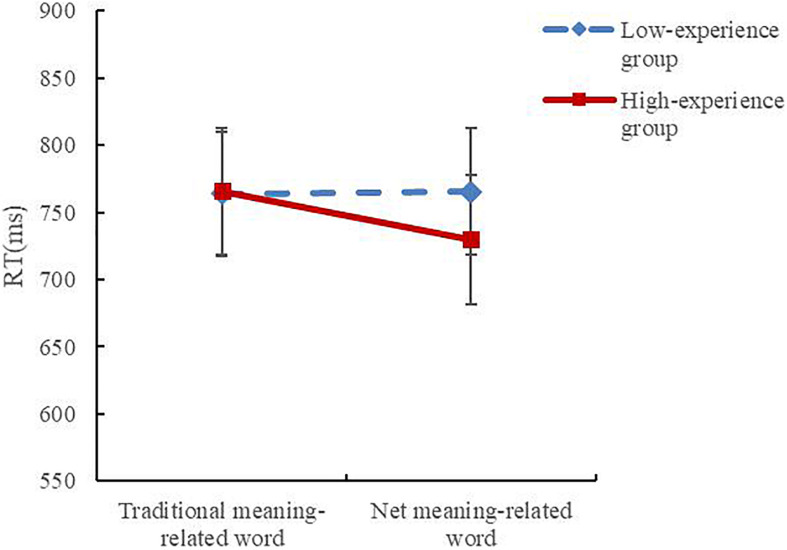
RTs of the two groups for two types of words (error bars depict the standard errors of the mean).

We explored the effects of the independent variables on RT via repeated-measures ANOVA. The results indicated that there was no significant main effect of net-speak experience or word type (*F*_1,57_ = 0.065, *p* = 0.799; *F*_1,57_ = 2.652, *p* = 0.109), but there was a nearly significant interaction between the two variables (*F*_1,53_ = 3.231, *p* = 0.078, $\eta_{\text{p}}^{2}$ = 0.054) (Figure 5).

The results of simple-effect tests indicated that the RT of the high-experience group for net-speak meaning-related words was significantly shorter than the RT for traditional meaning-related words (*F*_1,57_ = 5.77, *p* = 0.02, $\eta_{\text{p}}^{2}$ = 0.092), but for the low-experience group, there was no significant difference (*F*_1,57_ = 0.015, *p* = 0.904). The results showed that for the experienced participants, the net-speak meaning seemed to be activated slightly faster than the traditional meaning. However, for the low-experience group, both meanings were activated with the same speed.

### Discussion

In Experiment 2, we tested the RT and ACC of the semantic judgment of net-speak words with both traditional and net-speak meanings. The results showed that the high-experience group responded more quickly to the net-speak meaning, and there was no significant difference in the decision ACC for the two kinds of semantically related words. In addition, the ACC of the two kinds of semantic decisions was higher for the highly experienced participants than for the participants with low experience.

The results are inconsistent with the findings of most cognitive processing studies on textism in English. Most studies (Head et al., 2013a; Sinéad et al., 2015) pointed out that the processing of English net-speak words required more cognitive effort, possibly because of the additional activation of standard orthography. However, Ganushchak et al. (2012) found that for college students with rich net-speak experience, the link between the orthography and semantics of textisms was almost identical to that of standard words, because when a single word was used as a prime (e.g., gr8/great—good), the two primes had similar priming effects. This means that the link between the orthography and semantics of textisms may be direct and may not involve the mediator of standard orthography. This implies that net-speak experience strengthens the link between the orthography and semantics of net-speak words. Similar to their results, we found that net-speak experience contributed to the link between orthography and the two meanings of dual semantic net-speak words.

The results of the present study are different from the findings of most studies on textism in English, which suggested that the activation of the meaning of textisms requires more cognitive effort than the activation of the meaning of standard words. Our different results may be related to the characteristics of dual semantic Chinese net-speak words. The multiple meanings of ambiguous Chinese words are represented as separate entries (Shen and Li, 2016). Because of the identical orthography, participants may automatically activate both meanings of the dual semantic net-speak word simultaneously without additional orthographic activation, even in an online context. Users with more experience in net-speak use these net-speak words more frequently online, which strengthens their net-speak meanings. In addition, there is a metaphorical or metonymic relationship between the two meanings of a dual semantic net-speak word, and the use of the net-speak meaning may reinforce its traditional meaning. This may explain why the high-experience participants make better decisions on both meanings than the low-experience participants.

The low-experience group exhibited a similar performance in decisions about the two kinds of meaning-related words. We speculate that there are two reasons for this result. First, both meanings of the net-speak words were balanced in the stimuli calibration. Second, more importantly, dual semantic net-speak words may have become popular simply because their net-speak meanings are related to informal expressions. Since the low-experience group does not use them often online and may have few opportunities to use the standard meanings in formal text, they probably only know the basic meanings of these words rather than use them with high familiarity. This can perhaps be explicitly supported by the relatively low accuracy rates achieved by both groups (especially the low-experience group). In this way, we can also explain why the highly experienced group has an advantage in responding to both meanings, because their use of net-speak meanings simultaneously reinforces traditional meanings.

Therefore, it can be inferred that the use of net-speak may simultaneously strengthen the link between both meanings and the orthography of dual semantic net-speak words in the mental lexicon, making the link with the basic net-speak meaning stronger than that with the traditional meaning.

## General Discussion

Owing to the popularity of net-speak words among teenagers, there is concern that their frequent use will have a negative impact on teenagers’ standard language learning and cognition. However, a large number of studies in English have found a positive correlation between the use of textism and traditional literacy (Blom et al., 2017). When comparing the cognitive processing of net-speak with that of standard language, researchers found that English net-speak words have similar semantic processing characteristics as standard words (Ganushchak et al., 2010, 2012). Although it requires more cognitive resources for people to process net-speak words or sentences containing net-speak words (Berger and Coch, 2010; Head et al., 2012a, 2013a,b; Farrell and Lyddy, 2012; Sinéad et al., 2015), it has not been found that individuals with more experience in net-speak exhibit worse processing of standard words or sentences. These results prove that the relationship (synonymous with different forms) between English net-speak language and standard language is similar to that between a mother tongue and a second language (Berger and Coch, 2010).

However, in most cases, Chinese net-speak words are homographs of standard words. This means that compared with English language processing, the processing of Chinese net-speak words may have different characteristics. Indeed, this study used Go/No-Go and semantic decision tasks and found that there was a close correlation between the cognitive processing of net-speak words and net-speak experience. Although the decisions on net-speak words in the two groups were made more slowly than those on standard words, the high-experience group exhibited better processing of both net-speak words and standard words than the low-experience group (Experiment 1). Additionally, the high-experience group had higher decision ACC for the two meanings of the dual semantic net-speak words and faster activation of the net-speak meaning than the low-experience group, and the activation of traditional meaning was no slower than that of the low-experience group (Experiment 2).

The results of the two experiments showed that, similar to findings in the English context (Blom et al., 2017), the use of Chinese net-speak did not have negative effects on the cognition and understanding of standard words; on the contrary, it may contribute to the cognition of standard words. The difference between the present results and most findings from the English context is that the experience with Chinese net-speak may strengthen the links between orthography and both the net-speak meaning and traditional meaning of dual semantic net-speak words, and the strengthening of the net-speak meaning is more obvious. In this respect, the study of English net-speak did not show a closer connection between orthography and semantics for textisms than for standard words. For more experienced participants, the priming effects of net-speak words and standard words on meaning-related words were equivalent (Ganushchak et al., 2012), even though the priming effects of net-speak words were smaller than those of standard words (Head et al., 2013a). This means that net-speak experience in English does not threaten the link between standard orthography and semantics. This is related to the form of English net-speak words. When processing net-speak words, participants need to activate both net-speak orthography and standard orthography at the same time, while Chinese net-speak words do not require this.

To some extent, the results of this study supplemented the findings of Qingbai et al. (2017) that when processing a net-speak word in one sentence, the participants used more cognitive resources, regardless of the semantic consistency between the net-speak word and sentence. They speculated that this might be because the participants did not automatically process the net-speak words. Taking into consideration all the findings of this study, we can speculate that this may be due not only to the lower familiarity of net-speak words but also to the fact that standard sentences are provided before the net-speak words, which may lead to the participants’ reactive tendency. That is, the context of the standard sentence may prime standard orthography, semantics or phonology for the blank in the sentence, which may interfere with the processing of net-speak words in the blank. However, this study found that for a dual semantic net-speak word with no context, no additional cognitive resources are needed to decide its net-speak meaning. These results suggested that context is an important factor affecting the processing of dual semantic net-speak words.

In short, this study preliminarily proved that the use of net-speak does not interfere with the processing of standard words but may be beneficial to the cognition of standard Chinese. People’s concern is not so much about the use of net-speak as about the excessive use of the Internet. Future studies can use the ERP approach to explore the time process of semantic activation for dual semantic net-speak words and further confirm the specific time difference in the activation of net-speak meanings and traditional meanings in the high-experience group. One limitation to this study is that low-experience and high-experience participants were chosen based on their AFUNQ scores, and their performance on the tasks was based again on net-speak words partially selected from the AFUNQ, so there may have been a learning effect. Another limitation is that we did not pre-test the language levels of the high-experience and low-experience participants. Although these participants were students in key classes, there is no guarantee that the language levels of the two groups were equivalent. The possible difference in language level between the high-experience and low-experience groups could have contributed to the differences in the experimental results between the two groups.

## Data Availability Statement

The datasets generated for this study are available on request to the corresponding author.

## Ethics Statement

The studies involving human participants were reviewed and approved by the School of Education, Hunan University of Science and Technology. Written informed consent to participate in this study was provided by the participants’ legal guardian/next of kin.

## Author Contributions

JC and RL participated in the design of this study. SH carried out the study and collected the data. JC and SH drafted the manuscript. All the authors read and approved the final manuscript.

## Conflict of Interest

The authors declare that the research was conducted in the absence of any commercial or financial relationships that could be construed as a potential conflict of interest.
