# Supplementary material for: Does Net-Speak Experience Interfere With the Processing of Standard Words? Evidence From Net-Speak Word Recognition and Semantic Decisions
Source: Front Psychol. 2020 Aug 7;11:1932. doi: 10.3389/fpsyg.2020.01932 (PMC7427605; doi:10.3389/fpsyg.2020.01932)
Supplement: Supplementary file 1 [file Table_1.DOCX]

**Appendix A.** Adolescent Familiarity with and Use of Net-speak Questionnaire

The following words are popular words in net-speak. Please tick√on the number according to your familiarity with them.

| N0. | Net-word  (Net-meaning) | I had never heard of that | I heard of it but I don't know the exact meaning | I know the meaning but I haven't used it | Occasionally used it | Generally used it (sometimes) | Used it frequently (very often) |
| --- | --- | --- | --- | --- | --- | --- | --- |
| 1 | 表(Don't) | ① | ② | ③ | ④ | ⑤ | ⑥ |
| 2 | 闪(Dodge) | ① | ② | ③ | ④ | ⑤ | ⑥ |
| 3 | 萌(Likable) | ① | ② | ③ | ④ | ⑤ | ⑥ |
| 4 | 饿(I or me) | ① | ② | ③ | ④ | ⑤ | ⑥ |
| 5 | 偶(I or me) | ① | ② | ③ | ④ | ⑤ | ⑥ |
|  | | | | | | | |
| 6 | 奇葩(Freak) | ① | ② | ③ | ④ | ⑤ | ⑥ |
| 7 | 童鞋(Schoolmate) | ① | ② | ③ | ④ | ⑤ | ⑥ |
| 8 | 鸭梨(Stress) | ① | ② | ③ | ④ | ⑤ | ⑥ |
| 9 | 潜水(Stealth landing) | ① | ② | ③ | ④ | ⑤ | ⑥ |
| 10 | 备胎(Fallback partner) | ① | ② | ③ | ④ | ⑤ | ⑥ |
|  | | | | | | | |
| 11 | 青蛙(Ugly man) | ① | ② | ③ | ④ | ⑤ | ⑥ |
| 12 | 洗具(Comedy) | ① | ② | ③ | ④ | ⑤ | ⑥ |
| 13 | 杯具(Tragedy) | ① | ② | ③ | ④ | ⑤ | ⑥ |
| 14 | 节奏(Tendency) | ① | ② | ③ | ④ | ⑤ | ⑥ |
| 15 | 毛线(Negation or exclamation) | ① | ② | ③ | ④ | ⑤ | ⑥ |
|  | | | | | | | |
| 16 | 宝宝(I) | ① | ② | ③ | ④ | ⑤ | ⑥ |
| 17 | 小强(Black beetle) | ① | ② | ③ | ④ | ⑤ | ⑥ |
| 18 | 不造(Be ignorant of) | ① | ② | ③ | ④ | ⑤ | ⑥ |
| 19 | 惊呆(See a wolf) | ① | ② | ③ | ④ | ⑤ | ⑥ |
| 20 | 醉了(nothing to say) | ① | ② | ③ | ④ | ⑤ | ⑥ |
|  | | | | | | | |
| 21 | 任性(Acolasia) | ① | ② | ③ | ④ | ⑤ | ⑥ |
| 22 | 劈腿(Infidelity) | ① | ② | ③ | ④ | ⑤ | ⑥ |
| 23 | 果酱(Overpraise) | ① | ② | ③ | ④ | ⑤ | ⑥ |
| 24 | 黑线(Embarrassed) | ① | ② | ③ | ④ | ⑤ | ⑥ |
| 25 | 表叔(Corrupt officials) | ① | ② | ③ | ④ | ⑤ | ⑥ |
|  | | | | | | | |
| 26 | 沙发(The first post in forum ) | ① | ② | ③ | ④ | ⑤ | ⑥ |
| 27 | 板凳(The second post in forum) | ① | ② | ③ | ④ | ⑤ | ⑥ |
| 28 | 包子(Coward) | ① | ② | ③ | ④ | ⑤ | ⑥ |
| 29 | 玻璃(Faggotry) | ① | ② | ③ | ④ | ⑤ | ⑥ |
| 30 | 餐具(Calamity) | ① | ② | ③ | ④ | ⑤ | ⑥ |
|  | | | | | | | |
| 31 | 草根(Civilian) | ① | ② | ③ | ④ | ⑤ | ⑥ |
| 32 | 冲浪(Browse Network) | ① | ② | ③ | ④ | ⑤ | ⑥ |
| 33 | 大虾(Master-hand) | ① | ② | ③ | ④ | ⑤ | ⑥ |
| 34 | 真心() | ① | ② | ③ | ④ | ⑤ | ⑥ |
| 35 | 葱白(Worship) | ① | ② | ③ | ④ | ⑤ | ⑥ |
|  | | | | | | | |
| 36 | 稀饭(Like) | ① | ② | ③ | ④ | ⑤ | ⑥ |
| 37 | 盖楼(Thread) | ① | ② | ③ | ④ | ⑤ | ⑥ |
| 38 | 狗血(Baloney) | ① | ② | ③ | ④ | ⑤ | ⑥ |
| 39 | 火星(Ignorant) | ① | ② | ③ | ④ | ⑤ | ⑥ |
| 40 | 可爱(Pathetic) | ① | ② | ③ | ④ | ⑤ | ⑥ |
|  | | | | | | | |
| 41 | 马甲(ID online) | ① | ② | ③ | ④ | ⑤ | ⑥ |
| 42 | 打酱油(none of my business) | ① | ② | ③ | ④ | ⑤ | ⑥ |
| 43 | 白骨精(Office lady) | ① | ② | ③ | ④ | ⑤ | ⑥ |
| 44 | 纯净水(Jabberwocky) | ① | ② | ③ | ④ | ⑤ | ⑥ |
| 45 | 油菜花(Talented girl) | ① | ② | ③ | ④ | ⑤ | ⑥ |
|  | | | | | | | |
| 46 | 贤惠(Bum around) | ① | ② | ③ | ④ | ⑤ | ⑥ |
| 47 | 小虾(Newbie) | ① | ② | ③ | ④ | ⑤ | ⑥ |
| 48 | 碉堡(Freaking awesome) | ① | ② | ③ | ④ | ⑤ | ⑥ |
| 49 | 河蟹(Harmonious) | ① | ② | ③ | ④ | ⑤ | ⑥ |
| 50 | 感冒(Care a hang) | ① | ② | ③ | ④ | ⑤ | ⑥ |
|  | | | | | | | |
| 51 | 粉丝(Fans) | ① | ② | ③ | ④ | ⑤ | ⑥ |
| 52 | 切糕(Forced sale) | ① | ② | ③ | ④ | ⑤ | ⑥ |
| 53 | 山寨(Bogus products) | ① | ② | ③ | ④ | ⑤ | ⑥ |
| 54 | 围脖(Microblog) | ① | ② | ③ | ④ | ⑤ | ⑥ |
| 55 | 虾米(What) | ① | ② | ③ | ④ | ⑤ | ⑥ |
|  | | | | | | | |
| 56 | 节操(Baseline) | ① | ② | ③ | ④ | ⑤ | ⑥ |
| 57 | 赶脚(Feel) | ① | ② | ③ | ④ | ⑤ | ⑥ |
| 58 | 闷骚(Prudery) | ① | ② | ③ | ④ | ⑤ | ⑥ |
| 59 | 酱紫(Such) | ① | ② | ③ | ④ | ⑤ | ⑥ |
| 60 | 逗逼(Funny) | ① | ② | ③ | ④ | ⑤ | ⑥ |
|  | | | | | | | |
| 61 | 恨嫁(Want to get marry) | ① | ② | ③ | ④ | ⑤ | ⑥ |
| 62 | 趴体(Party) | ① | ② | ③ | ④ | ⑤ | ⑥ |
| 63 | 可耐(Loveliness) | ① | ② | ③ | ④ | ⑤ | ⑥ |
| 64 | 木油(None) | ① | ② | ③ | ④ | ⑤ | ⑥ |
| 65 | 躺枪(Innocent) | ① | ② | ③ | ④ | ⑤ | ⑥ |
|  | | | | | | | |
| 66 | 颜值(Facial attractiveness) | ① | ② | ③ | ④ | ⑤ | ⑥ |
| 67 | 吐槽(Complain) | ① | ② | ③ | ④ | ⑤ | ⑥ |
| 68 | 脑残(Brainless) | ① | ② | ③ | ④ | ⑤ | ⑥ |
| 69 | 男票(Boyfriend) | ① | ② | ③ | ④ | ⑤ | ⑥ |
| 70 | 骚年(Teens) | ① | ② | ③ | ④ | ⑤ | ⑥ |
|  | | | | | | | |
| 71 | 弱爆(Too weak) | ① | ② | ③ | ④ | ⑤ | ⑥ |
| 72 | 腹黑(a friendly-looking villain) | ① | ② | ③ | ④ | ⑤ | ⑥ |
| 73 | 酸爽(Comfortable) | ① | ② | ③ | ④ | ⑤ | ⑥ |
| 74 | 愤青(Young cynic) | ① | ② | ③ | ④ | ⑤ | ⑥ |
| 75 | 泪奔(Shed tears) | ① | ② | ③ | ④ | ⑤ | ⑥ |
|  | | | | | | | |
| 76 | 浮云(Unimportant things) | ① | ② | ③ | ④ | ⑤ | ⑥ |
| 77 | 逼格(High-class) | ① | ② | ③ | ④ | ⑤ | ⑥ |
| 78 | 屌丝(Loser) | ① | ② | ③ | ④ | ⑤ | ⑥ |
| 79 | 剩女(Spinster) | ① | ② | ③ | ④ | ⑤ | ⑥ |
| 80 | 友尽(The end of friendship) | ① | ② | ③ | ④ | ⑤ | ⑥ |
|  | | | | | | | |
| 81 | 鸡冻(Exciting) | ① | ② | ③ | ④ | ⑤ | ⑥ |
| 82 | 死党(Best friends) | ① | ② | ③ | ④ | ⑤ | ⑥ |
| 83 | 拼爹(Parental competition) | ① | ② | ③ | ④ | ⑤ | ⑥ |
| 84 | 半熟女(Young married woman) | ① | ② | ③ | ④ | ⑤ | ⑥ |
| 85 | 葛优躺(Lie effeminately in chair) | ① | ② | ③ | ④ | ⑤ | ⑥ |
|  | | | | | | | |
| 86 | 猴赛雷(Be something) | ① | ② | ③ | ④ | ⑤ | ⑥ |
| 87 | 高大上(Magnificent and classy) | ① | ② | ③ | ④ | ⑤ | ⑥ |
| 88 | 剁手党(Shopaholic) | ① | ② | ③ | ④ | ⑤ | ⑥ |
| 89 | 凤凰男(A man born in poverty but living in a city) | ① | ② | ③ | ④ | ⑤ | ⑥ |
| 90 | 孔雀女(City girl) | ① | ② | ③ | ④ | ⑤ | ⑥ |
|  | | | | | | | |
| 91 | 伤不起(Fragile) | ① | ② | ③ | ④ | ⑤ | ⑥ |
| 92 | 毁三观(Subverting tradition) | ① | ② | ③ | ④ | ⑤ | ⑥ |
| 93 | 笑抽了(Laugh wildly) | ① | ② | ③ | ④ | ⑤ | ⑥ |
| 94 | 矮矬穷(Poor, short and ugly) | ① | ② | ③ | ④ | ⑤ | ⑥ |
| 95 | 白富美(White, rich, and beautiful girl) | ① | ② | ③ | ④ | ⑤ | ⑥ |
|  | | | | | | | |
| 96 | 月光族(Paycheck to paycheck) | ① | ② | ③ | ④ | ⑤ | ⑥ |
| 97 | 蓝瘦香菇(Feel awful and want to cry) | ① | ② | ③ | ④ | ⑤ | ⑥ |
| 98 | 洪荒之力(All the strength) | ① | ② | ③ | ④ | ⑤ | ⑥ |
| 99 | 吃瓜群众(Onlooker) | ① | ② | ③ | ④ | ⑤ | ⑥ |
| 100 | 我想静静(I miss the girl named Jingjing) | ① | ② | ③ | ④ | ⑤ | ⑥ |
