# Supplementary material for: Does Net-Speak Experience Interfere With the Processing of Standard Words? Evidence From Net-Speak Word Recognition and Semantic Decisions
Source: Front Psychol. 2020 Aug 7;11:1932. doi: 10.3389/fpsyg.2020.01932 (PMC7427605; doi:10.3389/fpsyg.2020.01932)
Supplement: Supplementary file 2 [file Table_2.DOCX]

Appendix B. Net-words, Standard words and Pseudo words

|  | **Net-words** | **Pseudo words** | **Standard words** | **Pseudo words** |
| --- | --- | --- | --- | --- |
|  | 赶脚(Feel) | 绯守 | 战略(Strategy) | 驰册 |
|  | 节操(Baseline) | 参放 | 沙漠(Desert) | 材别 |
|  | 酱紫(Such) | 城祸 | 关系(Relation) | 探憾 |
|  | 逗逼(Funny) | 抱订 | 肩膀(Shoulder) | 益浅 |
|  | 颜值(Facial attractiveness) | 尾从 | 纪律(Discipline) | 节别 |
|  | 月光族(Paycheck to paycheck) | 定隙间 | 阴谋(Conspiracy) | 毁急 |
|  | 脑残(Brainless) | 赤浆 | 时候(Time) | 路匪功子 |
|  | 男票(Boyfriend) | 束底 | 明确(Explicit) | 子蚀 |
|  | 骚年(Teens) | 坑会 | 原理(Principle) | 妥序 |
|  | 蓝瘦香菇(Feel awful and want to cry) | 叫律讯山 | 困得不行(Sleepy) | 深修变努 |
|  | 酸爽(Comfortable) | 松私 | 限制(Limit) | 民专 |
|  | 愤青(Young cynic) | 心程 | 声音(Voice) | 摔漫 |
|  | 洪荒之力(All the strength) | 力出向本 | 实事求是(Seek truth from facts) | 天日始从 |
|  | 吃瓜群众(Onlooker) | 犯录报队 | 无产阶级(Proletariat) | 上续审失 |
|  | 葛优躺(Lie effeminately in chair) | 路功数 | 有时候(Sometimes) | 揭相开 |
|  | 剩女(Spinster) | 犯势 | 知道(Know) | 灼别 |
|  | 友尽(The end of friendship) | 夕抱 | 目标(Goal) | 租隔 |
|  | 高大上(Magnificent and classy | 乱毁手 | 教育(Education) | 备解 |
|  | 剁手党(Shopaholic) | 夜咽定 | 混凝土(Concrete) | 着教店 |
|  | 拼爹(Parental competition) | 起方 | 共产主义(Communism) | 方界房稿 |
|  | 弱爆(Too weak) | 冲奈 | 社会(Society) | 给径 |
|  | 伤不起(Fragile) | 专奉列 | 老人家(Elderly) | 权梁配 |
|  | 大咖(master) | 畅轴 | 问题(Question) | 订失 |
|  | 蛇精病(insane) | 空数无 | 面前(Before) | 探意 |
|  | 死党(Best friends) | 斥漫 | 预备(Ready) | 历投 |
|  | 毁三观(Subverting tradition) | 妥计身 | 调整(Adjust) | 重自 |
|  | 吐槽(Complain) | 圆匪 | 骄傲(Pride) | 惊侧 |
|  | 笑抽了(Laugh wildly) | 材急水 | 看见(See) | 业拍 |
|  | 矮矬穷(Poor, short and ugly) | 会检盆 | 差不多(Almost) | 报速风 |
|  | 白富美(White, rich, and beautiful girl) | 桌追 | 根据地(Base area) | 伤接动 |
|  | 逼格(High-class) | 典益 | 研究(Research) | 走瓦 |
|  | 屌丝(Loser) | 苗造 | 世界(World) | 品律 |
|  | 腹黑(a friendly-looking villain) | 屠局 | 思想(Idea) | 瓦犹 |
|  | 浮云(unimportant things) | 品认 | 发展(Development) | 力书 |
|  | 灰常(Very) | 节专 | 阶级(Class) | 令闪 |
|  | 鸡冻(Exciting) | 月开 | 国家(Country) | 采本 |
|  | 萌萌哒(Loveliness) | 力击招 | 法西斯(Fascism) | 只国壳 |
|  | 泪奔(Shed tears) | 倾伙 | 科学(Science) | 务领 |
|  | 闷骚(Prudery) | 劝妆 | 眼睛(Eye) | 悟设 |
|  | 高富帅(Tall, rich and handsome man) | 笔解云 | 手榴弹(Grenade) | 略保罗 |
