# Supplementary material for: Does Net-Speak Experience Interfere With the Processing of Standard Words? Evidence From Net-Speak Word Recognition and Semantic Decisions
Source: Front Psychol. 2020 Aug 7;11:1932. doi: 10.3389/fpsyg.2020.01932 (PMC7427605; doi:10.3389/fpsyg.2020.01932)
Supplement: Supplementary file 3 [file Table_3.DOCX]

**Appendix C.** Dual semantic Net-words and meaning-related words

|  | Net-words  (Dual meaning) | Traditional meaning  related words | Net-meaning related words |
| --- | --- | --- | --- |
| **1** | 杯具(cups/tragedy) | 茶水(tea) | 悲剧(cothurnus) |
| **2** | 备胎(spare/ fallback partner) | 汽车(car) | 陪衬(backup) |
| **3** | 潜水(underwater diving/ stealth landing) | 游泳(swimming) | 隐身(hiding) |
| **4** | 粉丝(vermicelli/fans) | 面条(noodles) | 拥护者(supporter) |
| **5** | 奇葩(wonderful flower/ freak) | 特别(special) | 另类(weird) |
| **6** | 稀饭(rice gruel/like) | 米粥(congee) | 喜欢(be fond of) |
| **7** | 表(meter/ don’t) | 手表(watch) | 不要(no) |
| **8** | 闪(flash/ dodge) | 电光(lightning) | 离开(leave) |
| **9** | 沙发(sofa/ The first post in forum) | 椅子(chair) | 第一(firstly) |
| **10** | 节奏(rhythm/ tendency) | 韵律(cadence) | 状态(status) |
| **11** | 毛线(woolen yarn/what) | 细绳(string) | 什么(what) |
| **12** | 冲浪(surf/ Browse network) | 戏水(paddle) | 网游(online game) |
| **13** | 小强(Xiao-qiang / Black beetle) | 人名(name) | 蟑螂(cockroach) |
| **14** | 马甲(waistcoat/ ID online) | 背心(vest) | 身份(identity) |
| **15** | 惊呆(amazing/see a wolf) | 吃惊(surprise) | 看傻(stunned) |
| **16** | 醉了(tipsy/nothing to say) | 喝醉(be in liquor) | 无语(speechless) |
| **17** | 任性(capricious/acolasia) | 随性(casual) | 放纵(arrogant) |
| **18** | 劈腿(trestle/infidelity) | 劈叉(the splits) | 出轨(betray) |
| **19** | 草根(grass root/civilian) | 植物(plant) | 平民(plebs) |
| **20** | 饿(hungry/I or me) | 饱(full up) | 我(I) |
| **21** | 狗血(dog blood/ baloney) | 淋头(shower) | 夸张(exaggerate) |
| **22** | 真心(sincere/really) | 实意(earnest) | 确实(true) |
| **23** | 感冒(cold/care a hang) | 生病(fall ill) | 感兴趣(interesting) |
| **24** | 碉堡(blockhouse/ freaking awesome) | 建筑(buildings) | 厉害(wonderful) |
| **25** | 山寨(cottage/bogus products) | 山中城寨(mountain village) | 高仿制品(replica) |
| **26** | 围脖(neckerchief/microblog) | 围巾(scarf) | 微博(microblog) |
| **27** | 虾米(shrimp/what) | 虾仁(shrimp meat) | 什么(what) |
| **28** | 我想静静  (I want to be quiet/ I miss the girl named Jingjing) | 我想安静一下  (I want to be quiet) | 我想那个叫静静的女孩  (I miss the girl named Jingjing) |
| **29** | 老司机  (experienced driver/ veteran) | 开车时间很长的司机  （experienced driver） | 经验丰富的高手  (veteran) |
| **30** | 小目标（small target/ ambitious goal） | 小计划(small plan) | 1个亿大目标  (Make 100 million yuan) |
